# ID 262 - Onde Estamos e Aonde Gostaríamos de Chegar na Ampliação do Programa Nacional de Triagem Neonatal no Sistema Único de Saúde

**DOI:** 10.5327/2237-9622.2025.v34s1.262

**Published:** 2025-11-25

**Authors:** Bruna Bento dos Santos, Alícia Dorneles Dornelles, Cecília de Oliveira Carvalho Faria, Júlia Cordeiro Milke, Arthur Alberti, Fernando Selistre, Ana Carolina Peçanha, Martina Wissmann, Aldenora Ximenes, Ida Vanessa Doederlein Schwartz

**Keywords:** doenças raras, triagem neonatal, deficiência de acil-CoA desidrogenase de cadeia média, homocistinúria clássica, economia e organizações de saúde

## Abstract

**Introdução:**

A triagem neonatal é fundamental para a detecção de doenças congênitas antes do aparecimento dos sintomas, permitindo intervenções que salvam vidas e melhoram a qualidade de vida de milhares de pessoas. A Lei n.º 14.154, de 26 de maio de 2021, alterou o Estatuto da Criança e do Adolescente (ECA), ampliando para 50 o número de doenças rastreadas no Programa Nacional de Triagem Neonatal (PNTN). No entanto, a Lei determina que o rol de doenças incluídas no PNTN será revisado periodicamente, com base em evidências científicas, priorizando as doenças com maior prevalência no País e aquelas com protocolo de tratamento aprovado e tratamento incorporado no Sistema Único de Saúde (SUS). Assim sendo, este estudo teve como objetivo identificar e descrever as recomendações da Comissão Nacional de Incorporação de Tecnologias no SUS (Conitec) sobre a ampliação do PNTN, bem como os desafios para a avaliação de incorporação dessas doenças.

**Método:**

Trata-se de um estudo descritivo, no qual foram realizadas buscas manuais por relatórios na base de recomendações da Conitec, a partir da data de publicação da Lei n.º 14.154/2021. Foram descritas as recomendações da Comissão, bem como as limitações descritas nos documentos.

**Resultados:**

Foram identificados dois relatórios de recomendação da Conitec que avaliaram a triagem neonatal de doenças incluídas na ampliação do PNTN, após a publicação da Lei n.º 14.754/2021: deficiência de acil-CoA desidrogenase de cadeia média (Relatório n.º 792) e homocistinúria clássica (Relatório n.º 816) por MS/MS. Para ambas as condições, o Comitê da Conitec deliberou por unanimidade recomendar a incorporação no SUS. As limitações descritas nos relatórios incluíram a certeza muito baixa no corpo de evidências, devido, em grande parte, à heterogeneidade entre as amostras dos estudos. Outro desafio identificado foi a avaliação independente do marcador utilizado na triagem de homocistinúria clássica, já que alguns apresentam um percentual de falso-negativo de até 50%. Por fim, os relatórios destacam que a inclusão de outras doenças genéticas no processo de triagem por MS/MS, permitindo a testagem simultânea de diversas condições, reduziria significativamente o impacto orçamentário estimado para cada tecnologia.

**Conclusão:**

Das 50 doenças previstas na ampliação do PNTN, apenas duas foram avaliadas pela Conitec, a partir de 2021. As limitações descritas nos relatórios de recomendação sugerem que o formato atualmente adotado pode não capturar na totalidade os verdadeiros benefícios clínicos e majorar o impacto orçamentário dessa ampliação. Para garantir o cumprimento integral da Lei n.º 14.154/2021, que prevê a revisão do rol com base em evidências científicas, é necessário adotar outras estratégias. Isso inclui investir em pesquisas baseadas em estudos-piloto da ampliação do PNTN e em avaliações econômicas que considerem a triagem simultânea das doenças.

